# Advances in Plant Taxonomy and Systematics

**DOI:** 10.3390/biology12040570

**Published:** 2023-04-09

**Authors:** Lorenzo Peruzzi

**Affiliations:** PLANTSEED Lab, Department of Biology, University of Pisa, Via Derna 1, 56126 Pisa, Italy; lorenzo.peruzzi@unipi.it

Systematics and taxonomy are basic sciences and are crucial for all applications dealing with living organisms [1]. Taxonomic classification schemes, sought by early scholars to reflect “natural systems” [2], are nowadays universally accepted to reflect actual systematic relationships among organisms.

Phylogenetic reconstructions based on molecular systematics have provided a stable classification system at class, order, and family levels for many plant groups (see, e.g., [3,4,5]). However, at the genus level, due to a lack of knowledge, many classifications are still unstable and a lot of taxonomic changes have been published [6], with species that are often recombined under different genera or synonymized with others. Taxonomy users, either in the scientific community or in wider society, perceive this as a relevant (and often not fully understood) problem [7,8]. However, these changes are the obvious consequence of an increase in systematic knowledge. In this respect, proposals and ideas to abandon Linnean taxonomy [9,10] have not been accepted so far. Fortunately, nomenclatural and taxonomic databases are becoming increasingly widespread and authoritative (see, e.g., [11]), meaning that this problem could be easily superseded.

At a microevolutionary level, an integrated taxonomic approach [12] using a number of independent lines of evidence [13] is needed to disentangle the complex systematic relationships among units of diversity [14].

Accordingly, on one side, there is the need to build sound taxonomic hypotheses using multiple lines of evidence (see, e.g., [15,16,17,18]); on the other hand, given the ongoing mass extinctions and the decline of taxonomists in academies [19,20], there is the need to speed up the recognition and description of biodiversity on earth. In this respect, citizen science could also be helpful [21], for instance in observing and capturing plant diversity with a coverage and frequency much higher than by just relying on academic scholars.

In the Special Issue “Advances in Plant Taxonomy and Systematics”, all the topics previously mentioned were addressed in 15 high-quality and original studies, involving plant groups and researchers from all continents. In particular, the phylogeny and biogeography of Mammilloid cacti from Mexico (Cactaceae, eudicots) [22], Euphorbiaceae subfam. Acalyphoideae (Malpighiales, eudicots) in the Americas [23], *Astragalus* sect. *Stereothrix* (Fabaceae, Fabales, eudicots) [24] and *Veronica* subg. *Pentasepalae* (Plantaginaceae, Lamiales, eudicots) [25] from Eurasia were addressed. Whole plastome comparison revealed phylogenetic relationships in *Crassula* (Crassulaceae, Saxifragales, eudicots) [26] and in the family Magnoliaceae (Magnoliales, early branching angiosperms) [27]. The systematics of polyploid and/or apomictic species complexes was studied in European groups such as the *Ranunculus auricomus* complex (Ranunculaceae, Ranunculales, eudicots) [28], the *Sorbus austriaca* complex (Rosaceae, Rosales, eudicots) [29], *Crocus* ser. *Verni* (Iridaceae, Asparagales, monocots) [30], and *Leucanthemum* (Asteraceae, Asterales, eudicots) [31]. Integrated taxonomic approaches were followed for the characterization of the Asian palm genus *Bentinckia* (Arecaceae, Arecales, monocots) [32], for addressing infraspecific variability in the European *Armeria arenaria* (Plumbaginaceae, Caryophyllales, eudicots) [33], and for describing a new species endemic to Italy in *Adonis* sect. *Adonanthe* (Rancunculaceae) [34]. A thorough morphometric study dealt with the taxonomically debated Mediterranean genus *Ophrys* (Orchidaceae, Asparagales, monocots), in which between 9 and over 400 species are recognized depending on the authors opinions [35], highlighting that “a serious challenge awaits writers of field guides to the European flora, as they struggle to summarise innumerable indistinguishable ‘species’ carved out of morphological continua”. Finally, images shared by citizen scientists to the iNaturalist platform and on Facebook were particularly helpful, as they aided the identification of four out of the nine Australian species of the carnivorous genus *Drosera* (Droseraceae, Caryophyllales, eudicots) [36].

## References

[1] Wheeler Q. (2020). A taxonomic renaissance in three acts. Megataxa.

[2] Lorch J. (1961). The Natural System in Biology. Philos. Sci..

[3] Chase M.W., Reveal J.L. (2009). A phylogenetic classification of the land plants to accompany APG III. Bot. J. Linn. Soc..

[4] Chase M.W., Christenhusz M.J.M., Fay M.F., Byng J.W., Judd W.S., Soltis D.E., Mabberley D.J., Sennikov A.N., Soltis P.S., The Angiosperm Phylogeny Group (2016). An update of the Angiosperm Phylogeny Group classification for the orders and families of flowering plants: APG IV. Bot. J. Linn. Soc..

[5] PPG I. (2016). A community-derived classification for extant lycophytes and ferns. J. Syst. Evol..

[6] Nimis P.L. (2001). A tale from Bioutopia. Nature.

[7] Garnett S.T., Christidis L. (2017). Taxonomy anarchy hampers conservation. Nature.

[8] Morrison W.R., Lohr J.L., Duchen P., Wilches R., Trujillo D., Mair M., Renner S.S. (2009). The impact of taxonomic change on conservation: Does it kill, can it save, or is it just irrelevant?. Biol. Cons..

[9] Casiraghi M., Galimberti A., Sandionigi A., Bruno A., Labra M. (2016). Life With or Without Names. Evol. Biol..

[10] Brower A.V.Z. (2020). Dead on arrival: A postmortem assessment of “phylogenetic nomenclature”, 20+ years on. Cladistics.

[11] Borsch T., Berendsohn W., Dalcin E., Delmas M., Demissew S., Elliott A., Fritsch P., Fuchs A., Geltman D., Guner A. (2020). World Flora Online: Placing taxonomists at the heart of a definitive and comprehensive global resource on the world’s plants. Taxon.

[12] Dayrat B. (2005). Towards integrative taxonomy. Biol. J. Linn. Soc..

[13] Turrill W.B. (1938). The expansion of taxonomy with special reference to spermatophyta. Biol. Rev. Biol. Proc. Camb. Phil. Soc..

[14] Oberprieler C. (2023). The Wettstein tesseract: A tool for conceptualising species-rank decisions and illustrating speciation trajectories. Taxon.

[15] De Giorgi P., Giacò A., Astuti G., Minuto L., Varaldo L., De Luca D., De Rosa A., Bacchetta G., Sarigu M., Peruzzi L. (2022). An integrated taxonomic approach points towards a single-species hypothesis for *Santolina* (Asteraceae) in Corsica and Sardinia. Biology.

[16] Liu L., Astuti G., Coppi A., Peruzzi L. (2022). Different chromosome numbers but slight morphological differentiation and genetic admixture among populations of the *Pulmonaria hirta* complex (Boraginaceae). Taxon.

[17] Mátis A., Malkócs T., Kuhn T., Laczkó L., Moysiyenko I., Szabó A., Bădărău A.S., Sramkó G. (2023). Hiding in plain sight: Integrative analyses uncover a cryptic *Salvia* species in Europe. Taxon.

[18] Giacò A., Varaldo L., Casazza G., De Luca D., Caputo P., Sarigu M., Bacchetta G., Sáez L., Peruzzi L. (2022). An integrative taxonomic study of *Santolina* (Asteraceae) from southern France and north-eastern Spain reveals new endemic taxa. J. Syst. Evol..

[19] Britz R., Hundsdörfer A., Fritz U. (2020). Funding, training, permits—The three big challenges of taxonomy. Megataxa.

[20] Wheeler Q. (2014). Are reports of the death of taxonomy an exaggeration?. New Phytol..

[21] Fontaine C., Fontaine B., Prévot A.-C. (2021). Do amateurs and citizen science fill the gaps left by scientists?. Curr. Opin. Insect..

[22] Delil A., Chincoya S.A., Felipe Vaca-Paniagua P.D., Solórzano S. (2023). Phylogenomics and biogeography of the Mammilloid clade revealed an intricate evolutionary history arose in the Mexican Plateau. Biology.

[23] Külkamp J., Riina R., Ramírez-Amezcua Y., Iganci J.R.V., Cordeiro I., González-Páramo R., Lara-Cabrera S.I., Baumgratz J.S.A. (2023). Systematics of Ditaxinae and related lineages within the subfamily Acalyphoideae (Euphorbiaceae) based on molecular phylogenetics. Biology.

[24] Bagheri A., Maassoumi A.A., Brassac J., Blattner F.R. (2023). Dated phylogeny of *Astragalus* section *Stereothrix* (Fabaceae) and allied taxa in the *Hypoglottis* clade. Biology.

[25] Doostmohammadi M., Bordbar F., Albach D.C., Mirtadzadini M. (2022). Phylogeny and historical biogeography of *Veronica* subgenus *Pentasepalae* (Plantaginaceae): Evidence for its origin and subsequent dispersal. Biology.

[26] Ding H., Han S., Ye Y., Bi D., Zhang S., Yi R., Gao J., Yang J., Wu L., Kan X. (2022). Ten plastomes of *Crassula* (Crassulaceae) and phylogenetic implications. Biology.

[27] Yang L., Tian J., Xu L., Zhao X., Song Y., Wang D. (2022). Comparative chloroplast genomes of six Magnoliaceae species provide new insights into intergeneric relationships and phylogeny. Biology.

[28] Hodač L., Karbstein K., Tomasello S., Wäldchen J., Bradican J.P., Hörandl E. (2023). Geometric morphometric versus genomic patterns in a large polyploid plant species complex. Biology.

[29] Hajrudinović-Bogunić A., Frajman B., Schönswetter P., Siljak-Yakovlev S., Bogunić F. (2023). Apomictic mountain whitebeam (*Sorbus austriaca*, Rosaceae) comprises several genetically and morphologically divergent lineages. Biology.

[30] Raca I., Blattner F.R., Waminal N.E., Kerndorff H., Ranđelović V., Harpke D. (2023). Disentangling *Crocus* series *Verni* and its polyploids. Biology.

[31] Oberprieler C., Ott T., Vogt R. (2023). Picks in the fabric of a polyploidy complex: Integrative species delimitation in the tetraploid *Leucanthemum* Mill. (Compositae, Anthemideae) representatives. Biology.

[32] Kadam S.K., Mane R.N., Tamboli A.S., Gavade S.K., Deshmukh P.V., Lekhak M.M., Choo Y.-S., Pak J.H. (2023). Cytogenetics, typification, molecular phylogeny and biogeography of *Bentinckia* (Arecoideae, Arecaceae), an unplaced Indian endemic palm from Areceae. Biology.

[33] Tiburtini M., Astuti G., Bartolucci F., Casazza G., Varaldo L., De Luca D., Bottigliero M.V., Bacchetta G., Porceddu M., Domina G. (2022). Integrative taxonomy of *Armeria arenaria* (Plumbaginaceae), with a special focus on the putative subspecies endemic to the Apennines. Biology.

[34] Conti F., Oberprieler C., Dorfner M., Schabel E., Nicoară R., Bartolucci F. (2023). *Adonis fucensis* (*A.* sect. *Adonanthe*, Ranunculaceae), a new species from the Central Apennines (Italy). Biology.

[35] Bateman R.M., Rudall P.J. (2023). Morphological continua make poor species: Genus-wide morphometric survey of the European bee orchids (*Ophrys,* L.). Biology.

[36] Krueger T., Robinson A., Bourke G., Fleischmann A. (2023). Small leaves, big diversity: Citizen science and taxonomic revision triples species number in the carnivorous *Drosera microphylla* complex (*D.* section *Ergaleium*, Droseraceae). Biology.

